# Supplementary material for: Extracellular sodium regulates fibroblast growth factor 23 (FGF23) formation
Source: J Biol Chem. 2023 Nov 21;300(1):105480. doi: 10.1016/j.jbc.2023.105480 (PMC10770535; doi:10.1016/j.jbc.2023.105480)
Supplement: Supporting Information [file mmc5.pdf]

## Supporting information

# Extracellular sodium regulates fibroblast growth factor 23 (FGF23) formation.

Zsuzsa Radvanyi[1,2†], Eun Jin Yoo[3†], Palanivel Kandasamy[4], Adrian Salas-Bastos[1], Sophie Monnerat[5,6], Julie Refardt[5,6], Mirjam Christ-Crain[5,6], Himeka Hayashi[7], Yasuhiko Kondo[7], Jonathan Jantsch[8,9], Isabel Rubio-Aliaga[10,2], Lukas Sommer[1], Carsten A. Wagner[10,2], Matthias A. Hediger[4], Hyug Moo Kwon[3], Johannes Loffing[1,2], Ganesh Pathare[1,2*]

[1]Institute of Anatomy, University of Zurich, Zurich, Switzerland; [2]Swiss National Centre of Competence in Research "Kidney Control of Homeostasis", Switzerland; [3]Department of Biological Sciences, Ulsan National Institute of Science and Technology, Ulsan, Republic of Korea; [4]Membrane Transport Discovery Lab, Department of Nephrology and Hypertension and Department of Biomedical Research, Inselspital, University of Bern, Bern, Switzerland; [5]Department of Endocrinology, Diabetology and Metabolism, University Hospital Basel, Basel, Switzerland; [6]Department of Clinical Research, University of Basel, Basel, Switzerland; [7]Department of Animal Sciences, Teikyo University of Science, Yamanashi, Japan; [8]Institute of Clinical Microbiology and Hygiene, University Hospital of Regensburg and University of Regensburg, Regensburg, Germany; [9]Institute for Medical Microbiology, Immunology, and Hygiene, and Center for Molecular Medicine Cologne (CMMC), University of Cologne, Cologne, Germany; [10]Institute of Physiology, University of Zurich, Zurich, Switzerland

[†]contributed equally to this work

***Correspondence author**: Ganesh Pathare, Institute of Anatomy, University of Zurich, Winterthurerstrasse 190, 8057, CH, Email: ganesh.pathare@anatomy.uzh.ch

**Keywords:** FGF23, extracellular-sodium, hyponatremia, NFAT5, bone and kidney

**This PDF file includes:**

Supplementary tables and figures

## Supplementary tables

| Cell Media | Osmolality (mOsm/Kg) |
|---|---|
| **Ctrl** | **300.7 ± 2.0** |
| **+NaCl (+20 mM)** | **345.7 ± 2.7** |
| **Mannitol (40 mM)** | **342.0 ± 1.5** |
| **Urea (40 mM)** | **342.3 ± 2.6** |

**Table S1.** Osmolality **±** SEM of the cell culture media used to study the effect of high extracellular [$Na^+$] (n=3, each group)

| Cell Media | Osmolality (mOsm/Kg) |
|---|---|
| **Ctrl** | **302.3 ± 3.7** |
| **-NaCl (-20 mM)** | **260.7 ± 1.5** |
| **-NaCl + Mannitol (40 mM)** | **302.7 ± 1.9** |
| **-NaCl + Urea (40 mM)** | **298.0 ± 1.7** |

**Table S2.** Osmolality **±** SEM of the cell culture media used to study the effect of low extracellular [$Na^+$] (n=3, each group)

| Nr | Age (y) | Sex | Weight (Kg) | Height (cm) | Serum $[Na^+]$ (mM) |
|---|---|---|---|---|---|
| **1** | 30 | m | 86 | 185 | 145 |
| **2** | 49 | f | 68.4 | 160 | 141 |
| **3** | 40 | m | 65.8 | 169 | 142 |
| **4** | 50 | m | 71.2 | 182 | 141 |
| **5** | 48 | m | 91.8 | 186 | 140 |
| **6** | 30 | m | 62.9 | 165 | 141 |

**Table S3.** Characteristics of the matching healthy control group.

| Nr | Age (y) | Sex | Weight (Kg) | Height (cm) | Serum $[Na^+]$ (mM) | Copeptin (pmol/L) | Etiology of Hyponatremia |
|---|---|---|---|---|---|---|---|
| 1 | 33 | m | 81.7 | 182 | 120 | 3.2 | SIADH (cause unknown) |
| 2 | 54 | m | 60 | 178 | 121 | 149 | Hypertonic translocation hyponatremia due to hyperglycemia |
| 3 | 47 | m | 70 | 170 | 123 | 110 | Hypovolemic Hyponatremia due to gastrointestinal fluid loss |
| 4 | 58 | m | 64 | missing | 123 | 11.9 | Hypovolemic Hyponatremia due to gastrointestinal fluid loss and mineralocorticoid deficiency |
| 5 | 55 | f | 55.5 | 152 | 121 | missing | Polydipsia (High Water Low Solute) |
| 6 | 55 | f | 35 | 170 | 123 | 2 | Polydipsia (High Water Low Solute) |

**Table S4.** Characteristics of the hyponatremic patients group.

## Supplementary figures

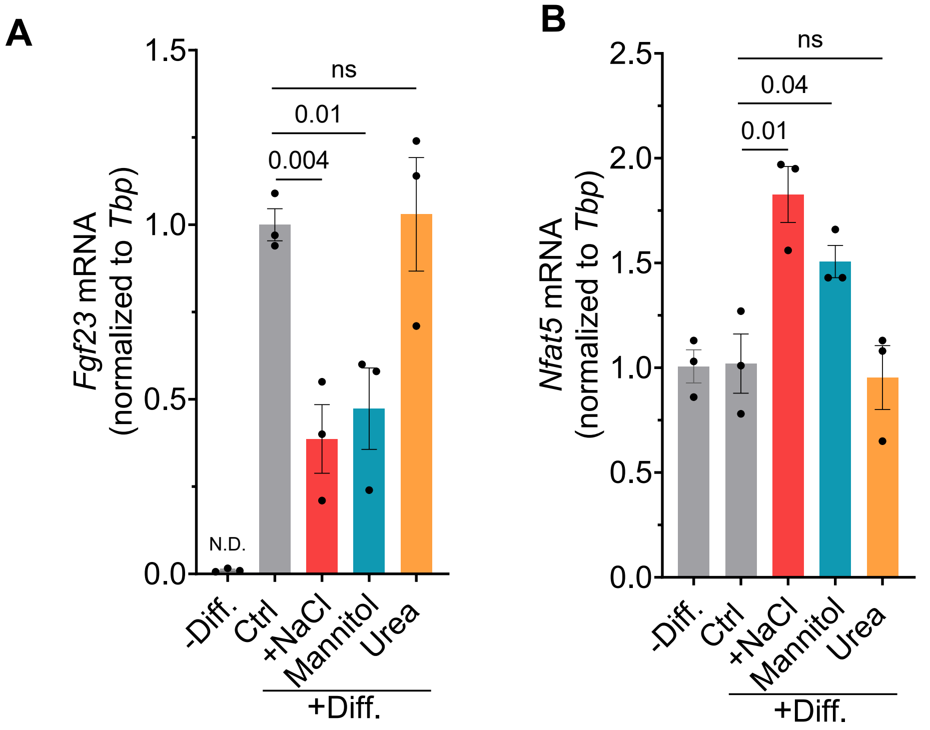


**Fig. S1. High NaCl suppresses FGF23 formation in MC3T3-E1 cells.** A) Fold change *Fgf23* mRNA levels; B) Fold change *Nfat5* mRNA levels, after treating 20 mM NaCl, 40 mM mannitol, and 40 mM urea for 24 h (n=3). *-Diff*. and *+Diff* indicate that cells were either not-differentiated or differentiated in the osteogenic media respectively.

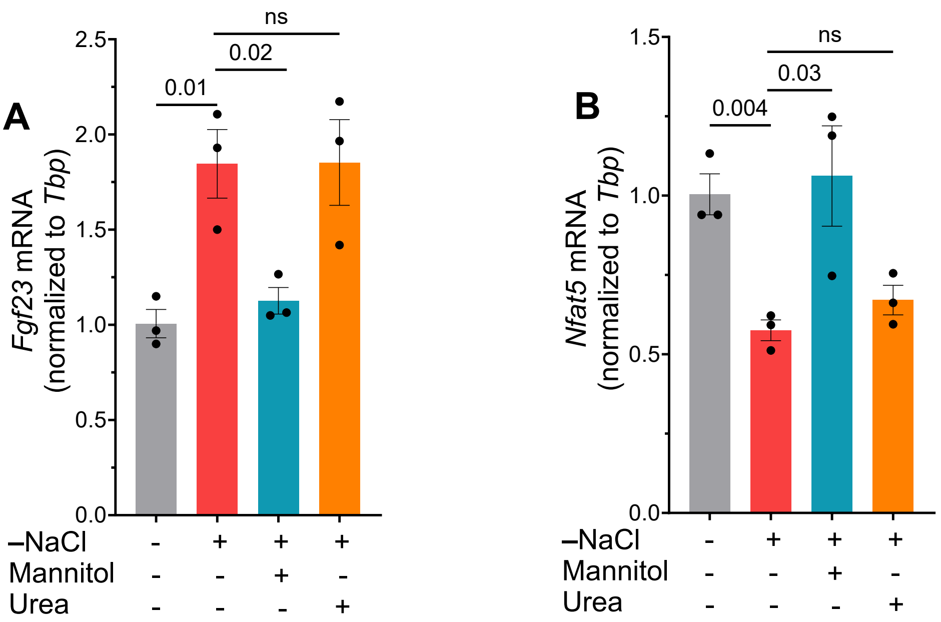


**Fig. S2. Hypotonicity elevates FGF23 formation in MC3T3-E1 cells.** A) Fold change *Fgf23* mRNA; B) *Nfat5* mRNA, after treating differentiated cells with -20 mM NaCl-deficient culture media. The osmolality was corrected by adding mannitol (40 mM) or urea (40 mM) for 24 h (n=3).

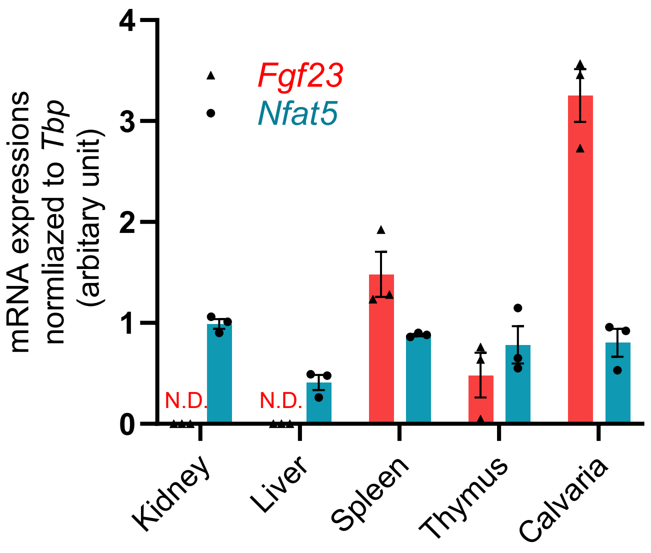


**Fig. S3.** The *Nfat5* and *Ffgf23* mRNA was measured by qRT-PCR in mouse kidneys, liver, spleen, thymus, and calvaria. Both *Nfat5* and *Ffgf23* mRNA were expressed in the spleen, thymus, and calvaria, while *Fgf23* mRNA was not detected (N.D) in the kidney and liver (n=3).

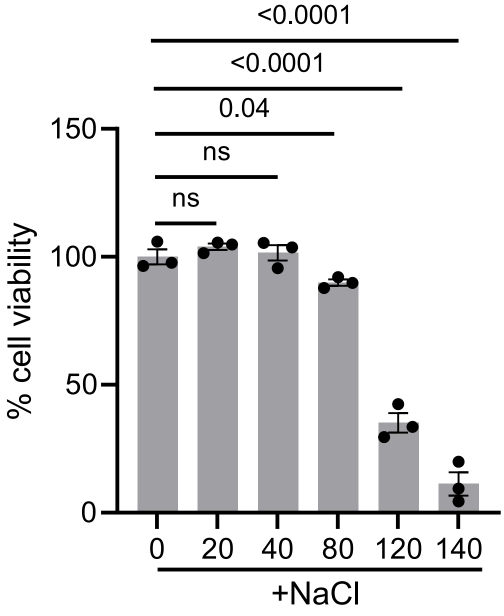


**Fig. S4.** Cell viability assessed through an MTT assay following the addition of +NaCl for 24 hours (n=3).

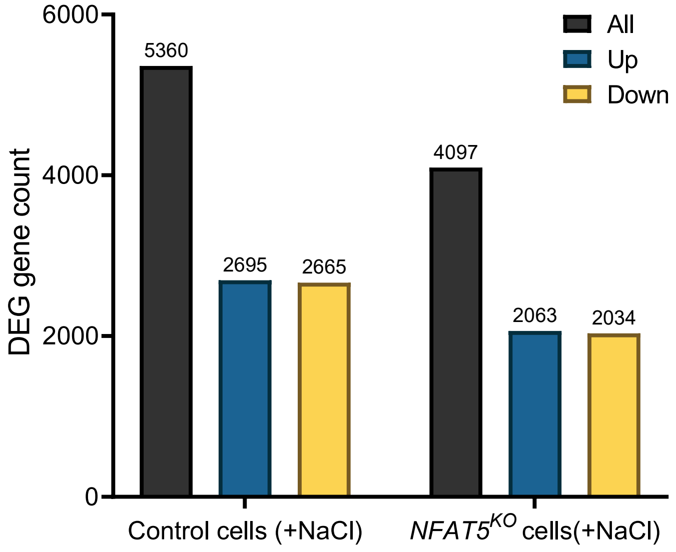


**Fig. S5.** Differentially expressed genes (DEG) count in control and *NFAT5*$^{KO}$ UMR-106 cells upon -NaCl vs. +NaCl treatment.

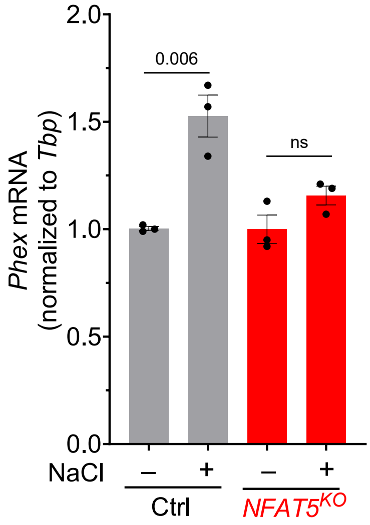


**Fig. S6.** *Phex* mRNA levels measured by qRT-PCR in control and *NFAT5*$^{KO}$ UMR-106 cells after -NaCl (-20 mM) vs. +NaCl treatment (+20 mM) for 24h (n=3, each group).

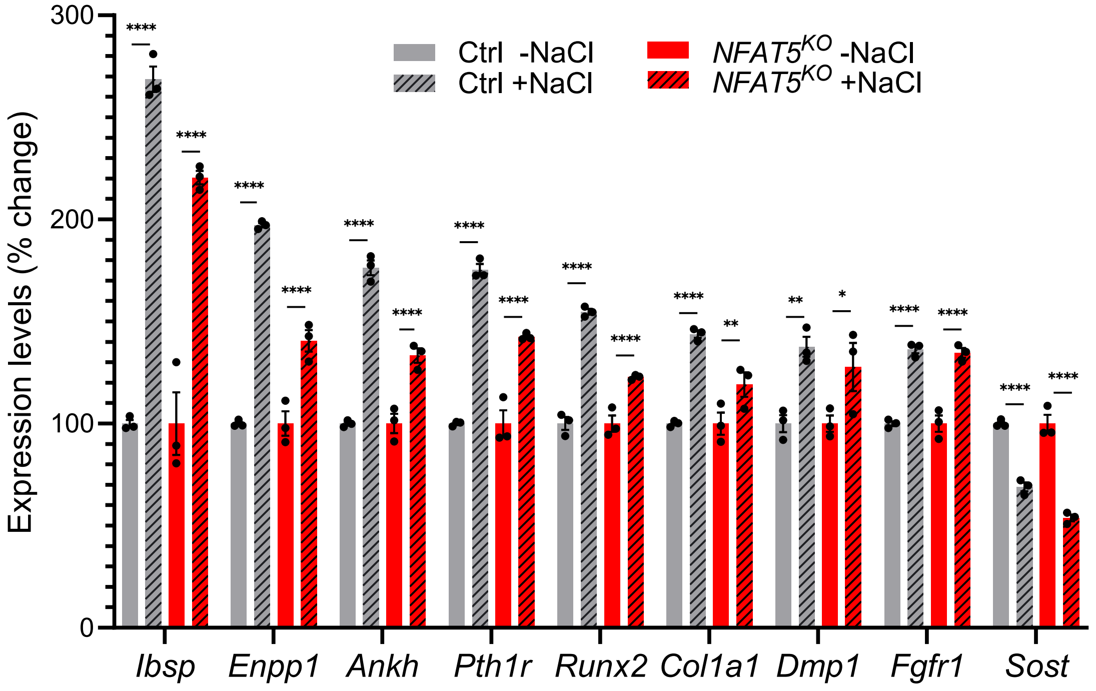


**Fig. S7.** The RNA-seq analysis resulted in DEG implicated in FGF23 regulation in both control and *NFAT5*$^{KO}$ UMR-106 cells. *$p \leq 0.05$, **$p < 0.01$, ***$p < 0.001$ and ****$p < 0.0001$

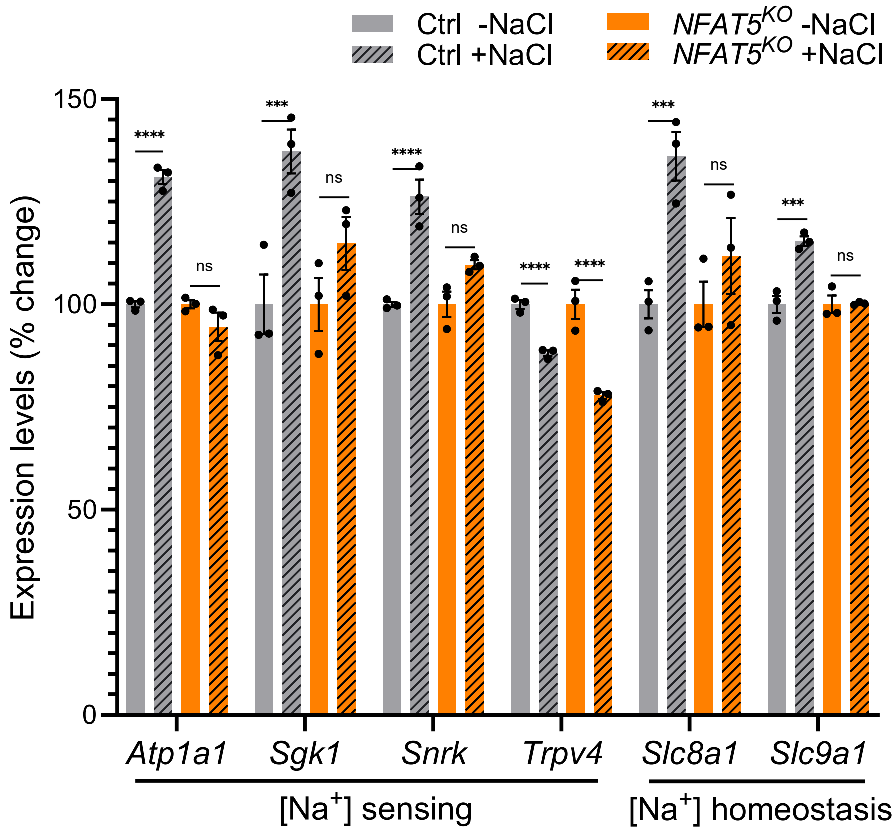


**Fig. S8.** The RNA-seq analysis resulted in DEG implicated in [$Na^+$] homeostasis in both control and *NFAT5*$^{KO}$ UMR-106 cells. (ns: not significant, $p > 0.05$, *$p \leq 0.05$, **$p < 0.01$, ***$p < 0.001$ and ****$p <$ 0.0001)

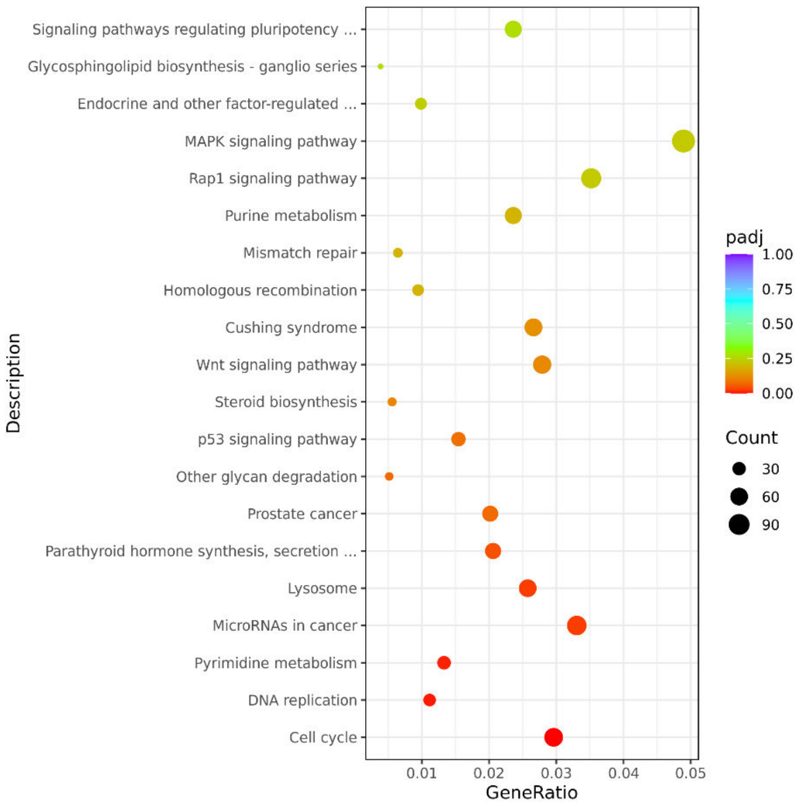


**Fig. S9. A)** KEGG pathways analysis in control UMR-106 cells upon +NaCl treatment.

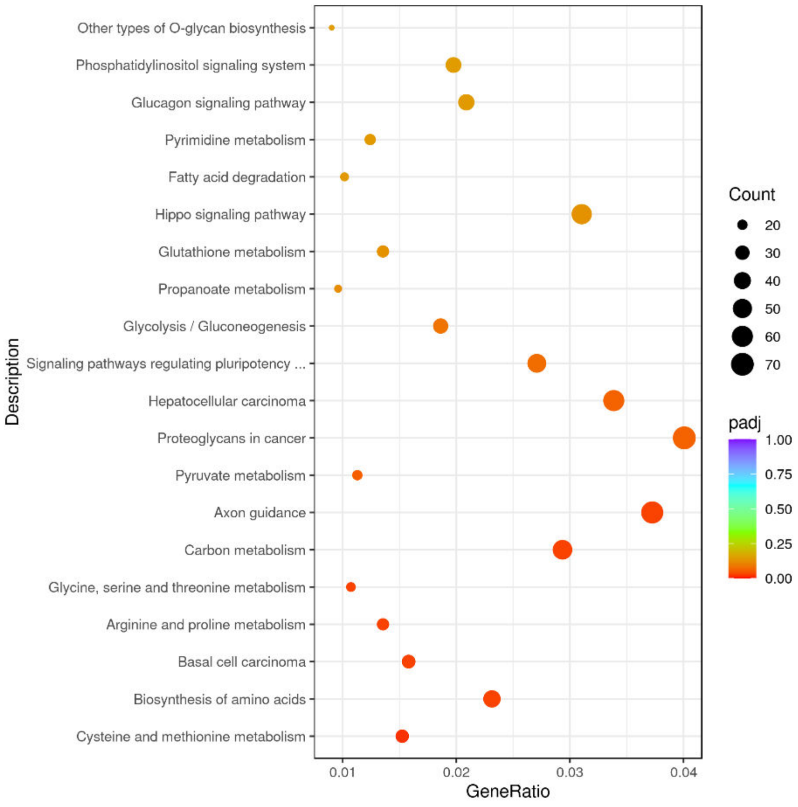


**Fig. S9. B)** KEGG pathways analysis in ***NFAT5$^{KO}$*** UMR-106 cells upon +NaCl treatment.

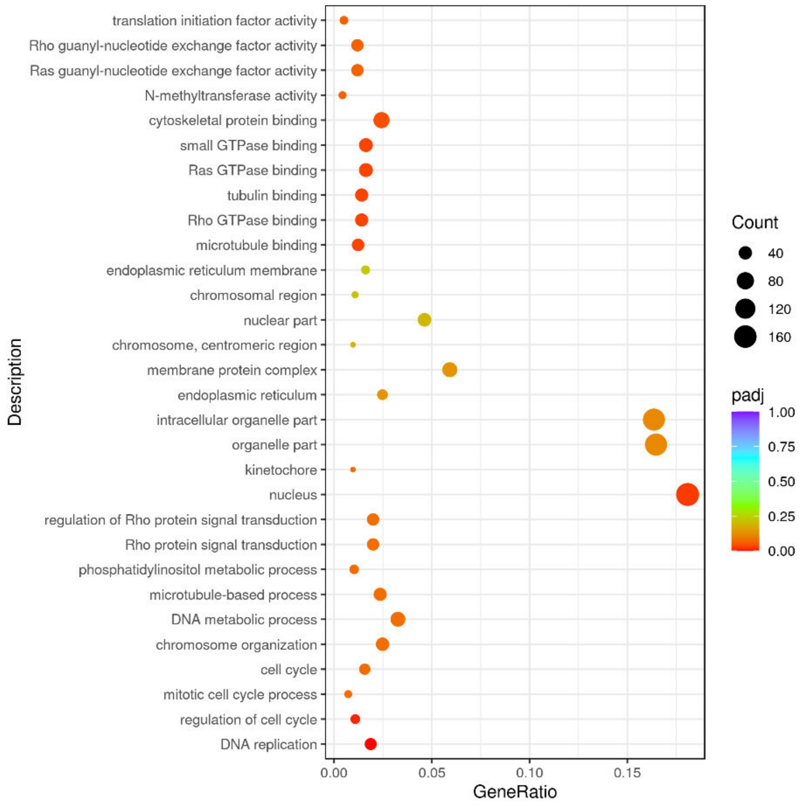


**Fig. S10. A)** GO pathways analysis in control UMR-106 cells upon +NaCl treatment.

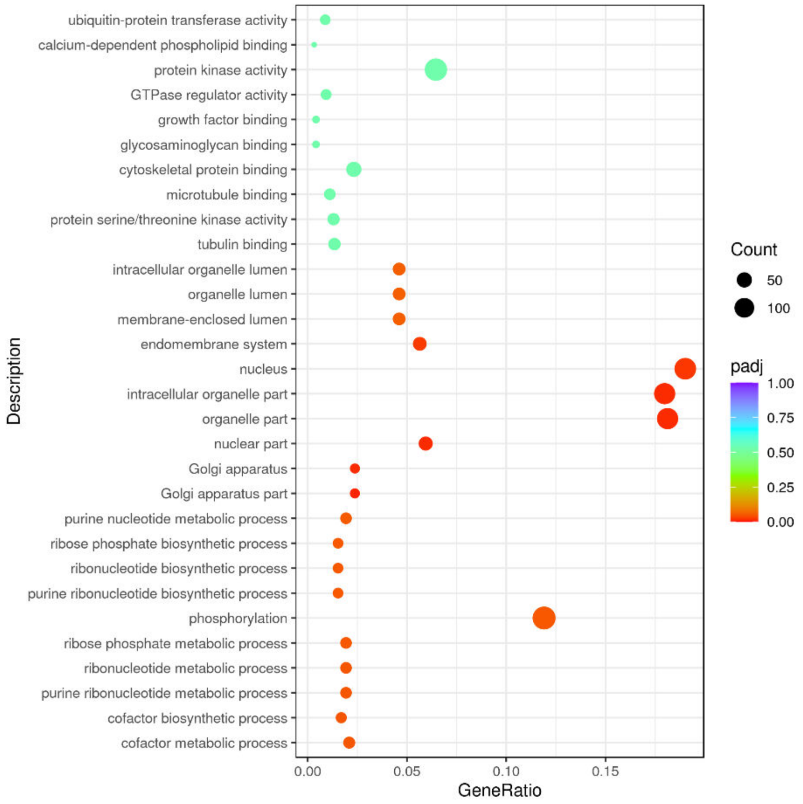


**Fig. S10. B)** GO pathways analysis in ***NFAT5<sup>KO</sup>*** UMR-106 cells upon +NaCl treatment.

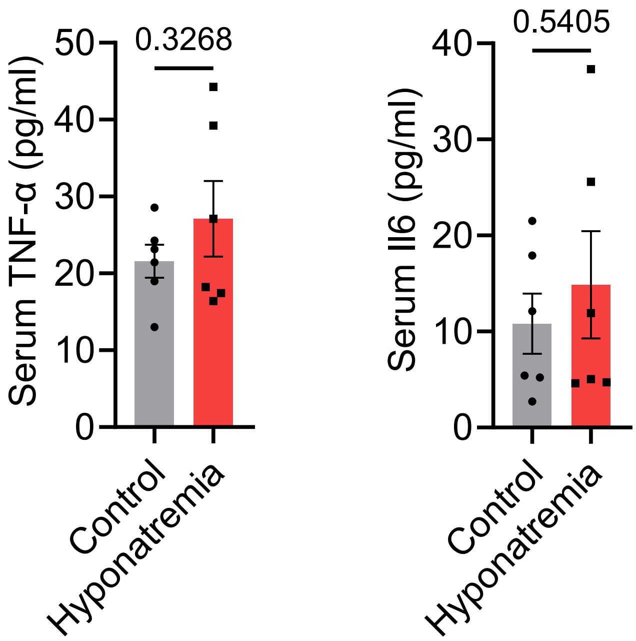


**Fig. S11**. Serum TNF-α and Il6 in healthy matching controls and hyponatremia patients (n=6, each group).
